# Relationship of prolonged global and regional central circulatory transit time with hemodynamics

**DOI:** 10.1186/1532-429X-17-S1-O5

**Published:** 2015-02-03

**Authors:** Jennifer M Conroy, Michael Passick, Jeannette McLaughlin, Jie J Cao

**Affiliations:** 1 Research Department, St. Francis Hospital, Roslyn, NY, USA

## Background

Normalized mean transit time (TT) in the left atrium has previously been shown to approximate left ventricular end diastolic pressure (LVEDP). In the present study, we characterized global and segmental central circulatory TT, in patients with and without systolic dysfunction, and delineated the relationship of TT to hemodynamics.

## Methods

Forty nine subjects undergoing clinically indicated right and left heart cardiac catheterization were prospectively recruited to undergo MRI in a 1.5T scanner. First pass perfusion using steady state free precession saturation recovery sequence was performed with gadolinium infusion at 0.01 mmol/kg. Global TT was defined as the time between the peaks of time intensity curves between the right atrium and the ascending aorta. Segmental TT included TT between right atrium to pulmonary artery (right heart TT), pulmonary artery to left atrium (pulmonary TT), or left atrium to ascending aorta (left heart TT). All TTs were normalized to heart rate. Multivariate regression analysis was performed to delineate the relationship of hemodynamic parameters measured during cardiac catheterization to TT. Receiver operating characteristic (ROC) analyses were performed to assess ability of global and segmental TT to predict elevated LVEDP.

## Results

Characteristics of the study group included average age of 62 years, LV ejection fraction (EF) 47±16%, and LVEDP 18 ± 9 mmHg. Global, pulmonary and left heart TTs were significantly prolonged in patients with elevated LVEDP compared to patients with LVEDP<15 mm Hg. Of the TTs assessed, global and pulmonary TT were strongly associated with hemodynamic abnormalities related to left heart failure in univariate analysis. In multivariate regression analysis adjusting right atrial pressure, pulmonary artery (PA) peak systolic and mean pressure, pulmonary vascular resistance, LVEDP and PA oxygen saturation, global TT was most significantly associated with LVEDP (p<0.001) whereas pulmonary TT was most significantly associated with LVEDP (p=0.009) and PA oxygen saturation (p=0.044). Using the ROC analysis, prolonged global TT and pulmonary TT were predictive of elevated LVEDP with an area under the curve (AUC) of 0.79 and 0.77, respectively. A cutpoint value of global TT ≥10.5 cardiac cycles and pulmonary TT ≥ 8.4 cardiac cycles were predictive of elevated LVEDP with sensitivity of 82% , 54% and specificity of 71% or 100%, respectively.

## Conclusions

Prolonged global and pulmonary TT were strongly associated with elevated LVEDP in patients with and without systolic dysfunction. Our findings suggest that global and pulmonary TT evaluation is promising in assessment of LVEDP elevation by MRI.

## Funding

N/A.

